# A Sulfated Glycosaminoglycan Linkage Region Is a Novel Type of Human Natural Killer-1 (HNK-1) Epitope Expressed on Aggrecan in Perineuronal Nets

**DOI:** 10.1371/journal.pone.0144560

**Published:** 2015-12-10

**Authors:** Keiko Yabuno, Jyoji Morise, Yasuhiko Kizuka, Noritaka Hashii, Nana Kawasaki, Satoru Takahashi, Shinji Miyata, Tomomi Izumikawa, Hiroshi Kitagawa, Hiromu Takematsu, Shogo Oka

**Affiliations:** 1 Department of Biological Chemistry, Human Health Sciences, Graduate School of Medicine, Kyoto University, Kyoto, 606–8507, Japan; 2 Department of Biological Chemistry, Graduate School of Pharmaceutical Sciences, Kyoto University, Kyoto, 606–8501, Japan; 3 Division of Biological Chemistry and Biologicals, National Institute of Health Sciences, Tokyo, 158–8501, Japan; 4 Department of Anatomy and Embryology Faculty of Medicine University of Tsukuba, Tsukuba, 305–8575, Japan; 5 Department of Biochemistry, Kobe Pharmaceutical University, Kobe, 658–8558, Japan; University of Leipzig, GERMANY

## Abstract

Human natural killer-1 (HNK-1) carbohydrate (HSO_3_-3GlcAβ1-3Galβ1-4GlcNAc-R) is highly expressed in the brain and required for learning and neural plasticity. We previously demonstrated that expression of the HNK-1 epitope is mostly abolished in knockout mice for GlcAT-P (*B3gat1*), a major glucuronyltransferase required for HNK-1 biosynthesis, but remained in specific regions such as perineuronal nets (PNNs) in these mutant mice. Considering PNNs are mainly composed of chondroitin sulfate proteoglycans (CSPGs) and regulate neural plasticity, GlcAT-P-independent expression of HNK-1 in PNNs is suggested to play a role in neural plasticity. However, the function, structure, carrier glycoprotein and biosynthetic pathway for GlcAT-P-irrelevant HNK-1 epitope remain unclear. In this study, we identified a unique HNK-1 structure on aggrecan in PNNs. To determine the biosynthetic pathway for the novel HNK-1, we generated knockout mice for GlcAT-S (*B3gat2*), the other glucuronyltransferase required for HNK-1 biosynthesis. However, GlcAT-P and GlcAT-S double-knockout mice did not exhibit reduced HNK-1 expression compared with single GlcAT-P-knockout mice, indicating an unusual biosynthetic pathway for the HNK-1 epitope in PNNs. Aggrecan was purified from cultured cells in which GlcAT-P and -S are not expressed and we determined the structure of the novel HNK-1 epitope using liquid chromatography/mass spectrometry (LC/MS) as a sulfated linkage region of glycosaminoglycans (GAGs), HSO_3_-GlcA-Gal-Gal-Xyl-R. Taken together, we propose a hypothetical model where GlcAT-I, the sole glucuronyltransferase required for synthesis of the GAG linkage, is also responsible for biosynthesis of the novel HNK-1 on aggrecan. These results could lead to discovery of new roles of the HNK-1 epitope in neural plasticity.

## Introduction

The extracellular matrix (ECM) plays vital roles in various physiological phenomena occurring in the central nervous system (CNS) [1–4]. In particular, perineuronal nets (PNNs), a specialized ECM structure surrounding neuronal cell bodies and proximal dendrites in the CNS, regulate neural plasticity [5, 6]. PNNs are composed of hyaluronan, chondroitin sulfate proteoglycans (CSPGs), link protein and tenascin-R [5–9] and ensheath parvalbumin (PV)-positive gamma-aminobutyric acid (GABA)-ergic interneurons and some pyramidal neurons [10–14]. Timing of PNN formation corresponds to the end of the critical period during which synaptogenesis, synaptic refinement and maturation of the nervous system occur [15–18], suggesting that formation of PNNs depends on completion or fixation of plastic change in neural activity. Previous reports demonstrated that cats reared in the dark with less sensory input showed delay in both the closure of the critical period and the emergence of PNNs in their brains [19, 20]. Additionally, sensory deprivation of whisker or facial vibrissae reduced the number of PNNs in rodent somatosensory cortex [21, 22]. These data strongly suggest that activity-dependent formation of PNNs has an important role in the regulation of neural plasticity.

Digestion of the chondroitin sulfate (CS) chain on CSPGs, a major component of PNNs, resulted in the reactivation of ocular dominance plasticity in adult mice [16]. Additionally, transgenic mice overexpressing chondroitin 6-sulfotransferase-1 (C6ST-1), a sulfotransferase involved in the 6-*O*-sulfation of GalNAc residues of the repeating disaccharide units in the CS chain, exhibited persistent ocular dominant plasticity in the adult brain, probably due to abnormal PNN morphology [23]. These data indicate that CSPGs, especially their CS chains, critically regulate neural plasticity in PNNs, but the detailed molecular mechanisms underlying the CS-dependent neural plasticity remain unclear.

To visualize PNNs, several monoclonal antibodies have been used; e.g., human natural killer-1 (HNK-1), 4F4 [24], VC1.1 [25] and Cat-315 [26], all of which react with the HNK-1 carbohydrate epitope, suggesting that the HNK-1 carbohydrate is involved in neural plasticity as a PNN component. The HNK-1 epitope is highly expressed in the CNS and its unique structure was determined to be a sulfated trisaccharide, HSO_3_-3GlcAβ1-3Galβ1-4GlcNAc-R [27]. We cloned and characterized two glucuronyltransferases (GlcAT-P and GlcAT-S) and a sulfotransferase (HNK-1ST) as key enzymes responsible for HNK-1 biosynthesis [28–30]. We also demonstrated that GlcAT-P-knockout (PKO) mice show a significant decrease in the HNK-1 epitope, resulting in reduced long-term potentiation (LTP) at the hippocampal CA1 region and impaired spatial learning [31]. These phenotypes are likely due to impairments in spine maturation and in cell surface retention of glutamate receptors in developing brains [32], indicating that the HNK-1 carbohydrate synthesized by GlcAT-P is required for the proper function of the mammalian CNS. Although HNK-1 monoclonal antibody (mAb) immunoreactivity was mostly abolished in the PKO mouse brain, some HNK-1 epitopes remained in the PNNs that co-localized with the lectin *Wisteria floribunda agglutinin* (WFA), another well-established PNN marker [31]. These results suggest that the HNK-1 epitope expressed in PNNs is synthesized by another glucuronyltransferase involved in neural plasticity such as GlcAT-S; however, its function, carrier protein and structure remain unclear.

In this study, we first explored a carrier protein of the GlcAT-P-irrelevant HNK-1 in PNNs and identified aggrecan. To examine its biosynthetic pathway, we generated GlcAT-S-knockout (SKO) mice as well as GlcAT-P and GlcAT-S double-knockout (DKO) mice and revealed that GlcAT-S is not responsible for its biosynthesis. Furthermore, using liquid chromatography/mass spectrometry (LC/MS) analysis, we found that a unique linkage region of the HNK-1 structure (HSO_3_-GlcA-Gal-Gal-Xyl-R) is expressed on aggrecan, which is likely synthesized by GlcAT-I but not by GlcAT-P and -S. Our study highlights new aspects of the HNK-1 epitope in PNNs.

## Materials and Methods

### Protein extraction from mouse brains

All the animal experiments were conducted according to the Fundamental Guidelines for Proper Conduct of Animal Experiments and Related Activities in Academic Research Institutions under the jurisdiction of the Ministry of Education, Culture, Sports, Science and Technology of Japan and approved by the Committees on Animal Experimentation of University of Tsukuba and Kyoto University. Whole brains dissected from 2- or 6-week-old mice were homogenized with 4 volumes of homogenizing buffer (20 mM Tris-HCl (pH 7.4) containing 150 mM NaCl, 1 mM EDTA and protease inhibitors (Nacalai Tesque)). The homogenates were centrifuged at 1,000 × g for 10 min to remove nuclei, followed by centrifugation at 105,000 × g for 60 min. The supernatants produced the Tris-buffered saline (TBS) soluble fractions. The pellets were rehomogenized with urea-containing buffer (10 mM Tris-HCl (pH7.4) containing 1 mM EDTA and 6 M urea) and then the homogenates were centrifuged at 105,000 × g for 60 min. The resulting supernatants were the urea-soluble fractions. The urea- and TBS-soluble fractions were combined and used as soluble fractions.

### Glycosidase digestion

Soluble fraction proteins (30 μg) from mouse brains were precipitated with ethanol and suspended with 20 to 50 μl of chondroitinase buffer (10 mM Tris-HCl (pH7.4), 30 mM sodium acetate, 50 mM EDTA and protease inhibitors), followed by incubation with a final concentration of 250 μU/ml of chondroitinase ABC (chABC) (Seikagaku Corporation) for 3 h at 37°C. The reaction mixtures were denatured with 0.5% sodium dodecyl sulfate (SDS) for 5 min at 100°C. To reduce the SDS concentration, the solution was diluted with 4 volumes of phosphate-buffered saline (PBS) containing 25 mM EDTA, 1.25% NP-40 and 1.25% 2-mercaptoethanol. Peptide *N*-glycosidase F (PNGase F, Roche Applied Science) was added at the final concentration of 20 mU/μl, followed by incubation for 16 h at 37°C.

### SDS-PAGE and western blotting

Proteins were separated in 3–10% gradient or 7% polyacrylamide gels and transferred to nitrocellulose membranes. After blocking with 5% nonfat dry milk in PBS containing 0.05% Tween 20, the membranes were incubated with primary antibodies, followed by horseradish peroxidase (HRP)-conjugated secondary antibodies. Protein bands were detected using Super Signal West Pico chemiluminescence reagent (Thermo Fisher Scientific), and the signal intensities were quantified using Image Gauge software (FUJIFILM). The following primary antibodies were used: HNK-1 monoclonal antibody (mAb) (a hybridoma cell line purchased from American Type Culture Collection, final concentration 4 μg/ml), M6749 mAb (kindly provided by Dr. H. Tanaka, Kumamoto University, final concentration 4 μg/ml), 6B4 mAb (developed as described previously [33], final concentration 4 μg/ml), Cat-315 mAb (Millipore, 1:500), anti-aggrecan polyclonal antibody (pAb) (Millipore, 1:500), anti-phosphacan pAb (antiserum was raised in rabbits against recombinant full-length phosphacan, which was expressed in and purified from COS-1 cells, final concentration 3.4 μg/ml), anti-NCAM mAb (kindly provided by Dr. K. Ono, Kyoto Prefectural University, clone H28, final concentration 0.7 μg/ml), anti-actin mAb (Millipore, 1:1000), anti-Fc pAb (Jackson ImmunoResearch, 1:1000), CS56 mAb (Sigma, 1:250) and anti-chondroitin sulfate A mAb (Seikagaku Corporation, clone IIH6, 1:250).

### Immunoprecipitation

Soluble fractions (600 μg) from mouse brains (PKO and DKO) were dialyzed against TBS using Slide-A-Lyzer Dialysis Cassette (Thermo Fisher Scientific) or precipitated with ethanol. After dialysis or ethanol-precipitation, proteins were suspended with 50 μl of chondroitinase buffer, followed by incubation with a final concentration of 250 μU/ml of chABC for 3 h at 37°C. The reaction mixtures were denatured with 0.5% SDS for 3 min at 100°C. To reduce the SDS concentration, the solution was diluted with 4 volumes of TBS containing 1% Triton X-100, followed by incubation with anti-aggrecan antibody (8 μg/ml final concentration), anti-phosphacan antibody (15 μg/ml final concentration) or normal rabbit IgG (Santa Cruz, 4 μg/ml final concentration) for 30 min at 4°C. The mixtures were then incubated with Protein G Sepharose (GE Healthcare) O/N at 4°C with gentle shaking. The beads were washed extensively with TBS containing 0.1% Triton X-100 and bound proteins were eluted by boiling in Laemmli sample buffer.

### Immunohistochemistry

Mice were deeply anesthetized and transcardially perfused with PBS and subsequently with PBS containing 4% paraformaldehyde. After cryoprotection with 30% sucrose, the tissues were sliced in 40-μm-thick sections using a microtome. Free-floating sections were incubated with 5 μg/ml primary antibodies (HNK-1 mAb, 6B4 mAb, Cat-315 mAb and aggrecan pAb) or biotinylated WFA lectin (Sigma, 1:400) diluted with PBS containing 3% bovine serum albumin for 2 h at room temperature, followed by incubation with Alexa Fluor conjugated secondary antibodies (Molecular Probes, 1:400) or Fluorescein AvidinD (VECTOR Laboratories, 1:400) for 1 h at room temperature. Sections were visualized under a Fluoview laser confocal microscope system, FV1000-D IX81 (Olympus).

### GlcAT-P (*B3gat1*) and GlcAT-S (*B3gat2*) deficient mice

**Fig 1:**
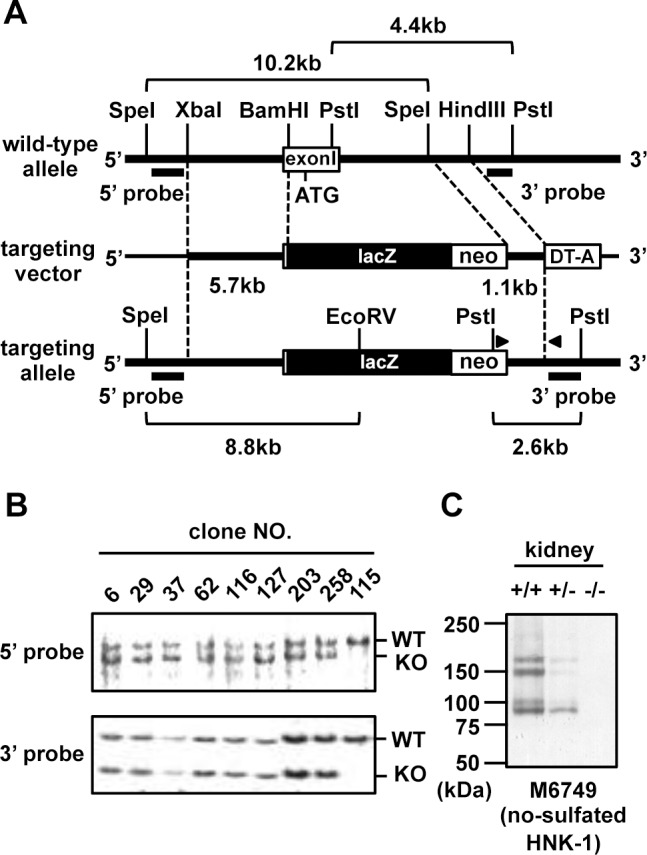
Generation of GlcAT-S-deficient mice. (A) Schematic diagram of the targeting construct. For targeted disruption of the mouse GlcAT-S gene (*B3gat2*), exon1 containing the initiation codon and transmembrane region were replaced with a neo/lacZ cassette. Hatched lines represent the fragments used for the construction of the targeting vector. PCR primers used for screening are shown in arrowheads. Black bars indicate probes for Southern blot analysis. (B) Southern blot analysis of targeted ES cell clones. Genomic DNA isolated from ES cell clones were digested with PstI or SpeI and EcoRV, blotted and then hybridized with the 3’ or 5’ probe, respectively. The expected sizes of the WT allele and targeted allele (KO) are indicated. The ES clones, 6, 29, 37, 62, 116, 127, 203 and 258 were positive when screening using PCR. ES clone 115 was used as a negative control. (C) Western blot analysis of SKO mice. The membrane proteins were prepared from 6-week-old mouse kidneys (WT; +/+, heterozygote; +/-, homozygote; -/-). A non-sulfated form of HNK-1 epitope was detected with M6749 mAb.

The method for generation of GlcAT-P gene (*B3gat1*)-deficient mice (PKO) was described previously [31]. GlcAT-S (*B3gat2*) gene-deficient mice (SKO) were produced as follows: genomic clone λ1011 harboring the exon1 of murine GlcAT-S, containing the initiation codon and transmembrane region was isolated from a murine 129/SvJλphage library [34]. Construction of the targeting vector is schematically represented in Fig 1A. A 5’ fragment (5.7 kb) containing a part of exon1 and 5’-end region and isolated by digestion using XbaI and BamHI was subcloned into pBluescript II SK(+) (pBS). A 3’ fragment (1.1 kb), a portion of the intron between exon1 and exon2, was digested with SpeI and HindIII, rendered blunt using Klenow enzyme and cloned into the EcoRV site of pBS. pCH110 (a gift from Dr. M. Asano, Kanazawa University) was digested with HindIII and BamHI and the β-galactosidase gene (3.7-kb fragment released) was religated with the vector containing the 5’ fragment. The neomycin resistance gene cassette in the vector pPGKneobpA [35] and the diphtheria toxin A (DT-A) gene cassette in vector pMC1DT-A [36] were introduced into the vector containing the 3’ fragment as positive and negative selection makers, respectively. The resulting plasmid was digested with NotI and ApaI, religated with the vector containing the 5’ fragment and β-galactosidase. The constructed targeting vector was transfected into ES cells and positive selection was performed using G418 (GIBCO). ES clones possessing the targeting allele from homologous recombination were selected using PCR and genome DNA was isolated from positive clones and analyzed using Southern blotting (Fig 1B). Targeting cell clones were injected into C57BL/6 blastocysts to obtain chimeric mice.

### Protein extraction from mouse kidney

Kidneys from 6-week-old wild-type (WT), GlcAT-S hetero (+/-) and homo (-/-) knockout mice were homogenized in 9 volumes of 20 mM Tris-HCl (pH 7.5) containing 150 mM NaCl, 1 mM EDTA and protease inhibitors at 4°C. The homogenates were centrifuged at 1,000 × g for 10 min at 4°C to remove nuclei. The supernatants were centrifuged at 105,000 × g for 1 h at 4°C. The resulting precipitates were used as membrane fractions.

### cDNA construction

Expression plasmid of aggrecan-Fc was constructed as follows. Two separate PCR reactions were performed to generate two overlapping gene fragments predicted to encode signal peptide, G1, CS2 and G3 domains (mini aggrecan) using human aggrecan cDNA obtained from the DNAFORM as a template. In the first PCR, the cDNA fragment containing signal peptide and G1 domain was amplified using a 5’-primer (5’- CGGGATCCGTCCAACTCTTCAAGGTGAACT-3’) containing an in-frame BamHI site (underlined) and a 3’-primer (5’-AAAGTCAGGCAGGCCTGTGTAGCAGATGGC-3’). In the second round of PCR, the cDNA fragment containing CS2 and G3 domains was amplified using a 5’-primer (5’-GCCATCTGCTACACAGGCCTGCCTGACTTT-3’) and a 3’-primer (5’-CGGGATCCGTGGGCTGTGCTGGGGCGGCT-3’) containing an in-frame BamHI site (underlined). The two PCR products were gel-purified and used as a template for a third PCR reaction containing the 5’-primer (5’- CGGGATCCGTCCAACTCTTCAAGGTGAACT-3’) containing an in-frame BamHI site and the 3’-primer (5’-CGGGATCCGTGGGCTGTGCTGGGGCGGCT-3’) containing an in-frame BamHI site. The final PCR fragment was digested with BamHI and ligated into pcDNA3.1-myc/His vector (Invitrogen) predigested with BamHI to obtain pcDNA3.1/mini aggrecan. The subcloning of the mini aggrecan into pEF-Fc mammalian expression vector was performed as follows: to insert a splicing donor signal, a cDNA fragment encoding a C-terminal region of aggrecan was amplified using PCR with a sense primer 5’-GCATCTAGAAATTGAGTCCTCAAG-3’ (endogenous XbaI site, underlined) and an antisense primer 5’-CCGTCTAGAGGATCCACTCACCGTGGGCTGTGCTGGGGCGGCTCCT-3’ (XbaI site, BamHI site and a splicing donor signal GTGAGT, underlined) using pcDNA3.1/mini aggrecan as a template. After digestion with XbaI, the product was ligated into pcDNA3.1/mini aggrecan previously digested with XbaI. Subsequently, the resultant plasmid was digested with BamHI and the aggrecan fragment was ligated into the pEF-Fc empty vector previously digested with BamHI. Expression plasmid of phosphacan-myc-Fc, myc-Fc-tagged full-length rat phosphacan, was described previously [37].

### Cell culture and transfection

COS-1 cells were purchased from American Type Culture Collection (ATCC CRL-1650). COS-1 cells were maintained in Dulbecco’s modified Eagle’s medium supplemented with 10% fetal bovine serum at 37°C until 50–70% confluency. For transfection, cells were plated on 175-cm^2^ culture flasks. Cells were transfected with the expression plasmids using X-tremeGENE HP DNA Transfection Reagent (Roche Applied Science) according to the manufacturer’s protocol. After 5–6 h of transfection, the culture medium was replaced with Ajinomoto serum-free Medium 104 (Ajinomoto). Cells were incubated for an additional 3 days to produce culture medium containing secreted proteins.

### Purification of recombinant aggrecan-Fc

Recombinant aggrecan-Fc was purified from the culture medium using Protein G Sepharose and eluted with 50 mM diethylamine pH 11.5 containing 150 mM NaCl. The eluate was immediately neutralized with 0.5 M NaH_2_PO_4_.

### Quantification of chondroitin sulfate

The purified recombinant aggrecan-Fc was subjected to gel filtration on a PD-10 column (GE Healthcare). The flow-through fractions were collected and digested with 5 mIU of chABC (Seikagaku Corporation) in 60 mM sodium acetate, 50 mM Tris-HCl (pH 8.0) for 12 h at 37°C. The digests were derivatized with a fluorophore, 2-aminobenzamide, and then analyzed by anion-exchange high-performance liquid chromatography (SLC-10A, Shimadzu) on a PA-03 column (YMC). Identification and quantification of the resulting disaccharides were achieved by comparison with chondroitin sulfate-derived authentic unsaturated disaccharides (Seikagaku Corporation), as described previously [38].

### LC/MS glycan analysis

The purified recombinant aggrecan-Fc treated with chABC and PNGase F to remove chondroitin sulfate and *N*-glycans was precipitated with ethanol and then lyophilized. *O*-glycans were released by hydrazinolysis and pyridylaminated according to a procedure described previously [37]. PA-labeled *O*-linked glycans were separated on a graphitized carbon column (150 × 0.075 mm, 5 μm; Chemicals Evaluation and Research Institute) at a flow rate of 300 nl/min in a Paradigm MS4 HPLC system (Michrom BioResources). The mobile phases were 1 mM ammonium acetate containing 2% acetonitrile (A buffer) and 1 mM ammonium acetate containing 80% acetonitrile (B buffer). The glycans were eluted with a linear gradient of 2–65% of B buffer for 50 min. MS was performed using an Orbitrap Elite Mass Spectrometer (Thermo Fisher Scientific). The electrospray voltage for MS was -2.5 kV in the negative ion mode and the collision energy was 25% for the MS/MS and MS/MS/MS experiments. Mass spectra were acquired by selected ion monitoring (SIM) mode. The resolution was 60,000 and the scan range was *m/z* 782.5–832.5.

## Results

### Aggrecan is an HNK-1 carrier protein in PKO mice

In PKO mouse brain, HNK-1 epitope was mostly abolished except for PNNs, indicating that it is suitable to analyze PKO mice to characterize HNK-1 carrier protein in PNNs. Previous reports demonstrated that HNK-1 epitope was expressed on aggrecan in the adult mouse brain [39] and especially in PNNs [26, 40]. Therefore, we examined whether the carrier protein of GlcAT-P-irrelevant HNK-1 epitope was also aggrecan or not.

**Fig 2:**
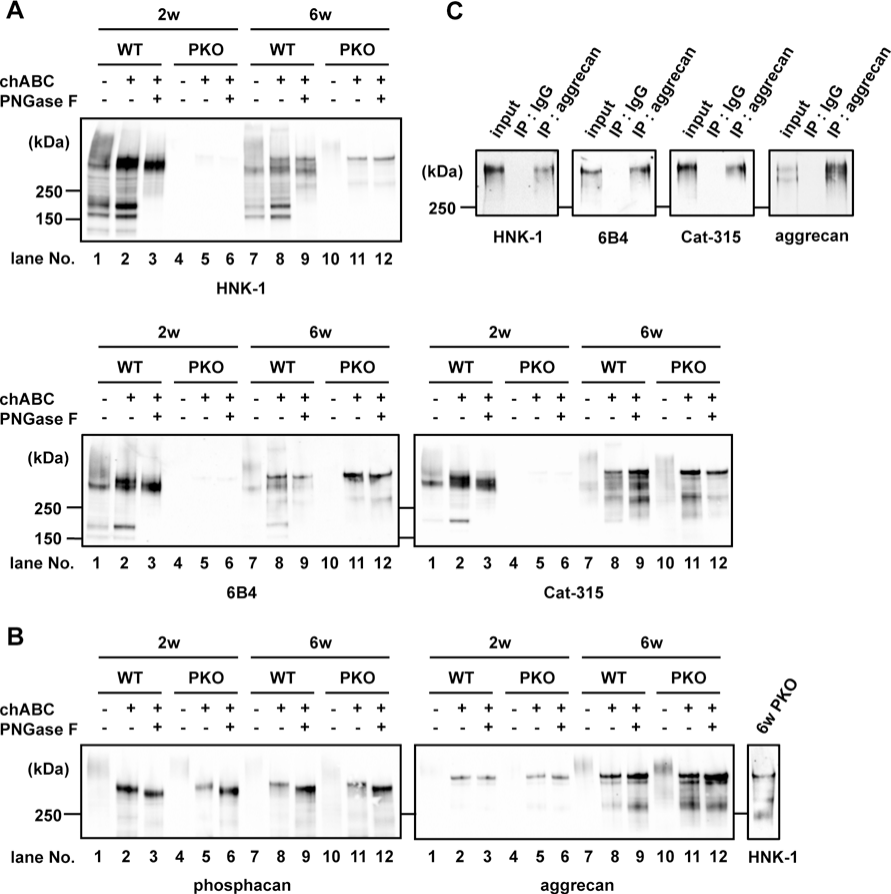
HNK-1 carbohydrate epitope expressed in GlcAT-P-deficient mice. (A, B) Soluble fractions prepared from 2- and 6-week-old mouse brains (WT and GlcAT-P-deficient mice) were treated with chABC with or without PNGase F, subjected to SDS-PAGE and then blotted using anti-HNK-1 mAb, 6B4 mAb, Cat-315 mAb, phosphacan pAb and aggrecan pAb. To compare the molecular weight of HNK-1 immunoreactive band with aggrecan or phosphacan, an HNK-1 mAb blot of 6-week-old PKO mice treated with chABC and PNGase F is shown on the right of the aggrecan panel. (C) Using urea-soluble fractions from 6-week-old PKO mouse brains (input), aggrecan was immunoprecipitated using aggrecan pAb (IP: aggrecan) or normal rabbit IgG (IP: IgG). The precipitated aggrecan was subjected to SDS-PAGE and western blotting with HNK-1, 6B4, Cat-315 and aggrecan antibodies.

To characterize the carrier protein of the HNK-1 epitope in PNNs in PKO mouse brain, brain soluble fractions including major PNN components were prepared from 2-week-old (PNN-unformed) and 6-week-old (PNN-formed) wild type (WT) and PKO mice (Fig 2A). Consistent with our previous report, all HNK-1 immunoreactive bands detected in WT mice had disappeared in 2-week-old PKO mice (Fig 2A, *HNK-1 panel*, *lanes 1 to 6*) [32, 37], indicating that GlcAT-P is dominant for HNK-1 synthesis in the developing brain. In contrast, after PNN formation was completed, a faint smear band over 250 kDa was detected with HNK-1 mAb in 6-week-old PKO mice (Fig 2A, *HNK-1 panel*, *lane 10*). Previous reports for the developing mouse brain showed that an HNK-1 carrying smear protein over 250 kDa was a CSPG, phosphacan, and was converged after chABC treatment [37, 39, 40, 41] (Fig 2A, *HNK-1 panel*, *lane 2*). This suggests the smear pattern of the band in the 6-week-old PKO mice was also derived from large CS chains. The molecular weight of the HNK-1-positive band in 6-week-old PKO mice shifted down after the removal of the CS chain with chABC. (Fig 2A, *HNK-1 panel*, *lane 11*), demonstrating the HNK-1 carrier protein in the 6-week-old PKO mice was a CSPG. In contrast to *N*-glycan-linked HNK-1 epitopes on many other HNK-1 carrier proteins such as GluA2, tenascin-R, NCAM, P0 and MAG in the CNS [32, 42, 43], removal of *N*-glycans with PNGase F did not reduce the HNK-1 signal in 6-week-old PKO mice (Fig 2A, *HNK-1 panel*, *lane 12*). This suggests that the HNK-1 epitope attached to the CSPG in 6-week-old PKO mice is present on a unique core glycan structure. The HNK-1-carrying molecule in 6-week-old PKO mice was also detected using two other HNK-1-recognizing antibodies, 6B4 and Cat-315 [26, 33, 37, 39, 40, 41] (Fig 2A, *6B4 and Cat-315 panels*), and the signals for this protein in 6-week-old WT mice appeared to be stronger than those for the other HNK-1-carrying molecules, which is consistent with the previous report by Dino et al. [39]. This indicates these two HNK-1-related mAbs react with HNK-1 epitopes with different specificities from HNK-1 mAb and prefer the unique HNK-1 epitope on CSPG.

Considering the molecular weights of the PNN component CSPGs (aggrecan, phosphacan, versican, brevican and neurocan) [5–9], aggrecan and phosphacan were the most feasible HNK-1 carrier candidates in the 6-week-old PKO mice. To test this possibility, mouse brain soluble fractions were immunoblotted for phosphacan and aggrecan after chABC and PNGase F treatment (Fig 2B). The molecular weight of aggrecan was similar to that of HNK-1, which was minimally affected by digestion with PNGase F in 6-week-old PKO mice (Fig 2B, *aggrecan panel*, *lane 12 and HNK-1 panel*). In contrast, the molecular weight of phosphacan and its larger downshift after PNGase F digestion were different from HNK-1 (Fig 2B, *phosphacan panel*, *lane 12*). These results indicate that aggrecan is the HNK-1 carrier protein in the 6-week-old PKO mouse brains.

To further confirm this assumption, we immunoprecipitated aggrecan from brain urea soluble fractions of 6-week-old PKO mice and then immunoblotted the precipitated aggrecan using HNK-1, 6B4, Cat-315 and anti-aggrecan antibodies. All three HNK-1 recognizing mAbs reacted with immunoprecipitated aggrecan; slightly higher reactivity with 6B4 and Cat-315 than HNK-1 mAb (Fig 2C) was observed, which was similar to the PKO soluble fraction (Fig 2A). Based on these results and the findings from other groups [26, 39, 40], in the 6-week-old mouse brain, we concluded that aggrecan was the carrier CSPG for the unique type of HNK-1 epitope generated in a GlcAT-P-independent manner.

### A unique HNK-1 epitope on aggrecan is located in PNNs

**Fig 3:**
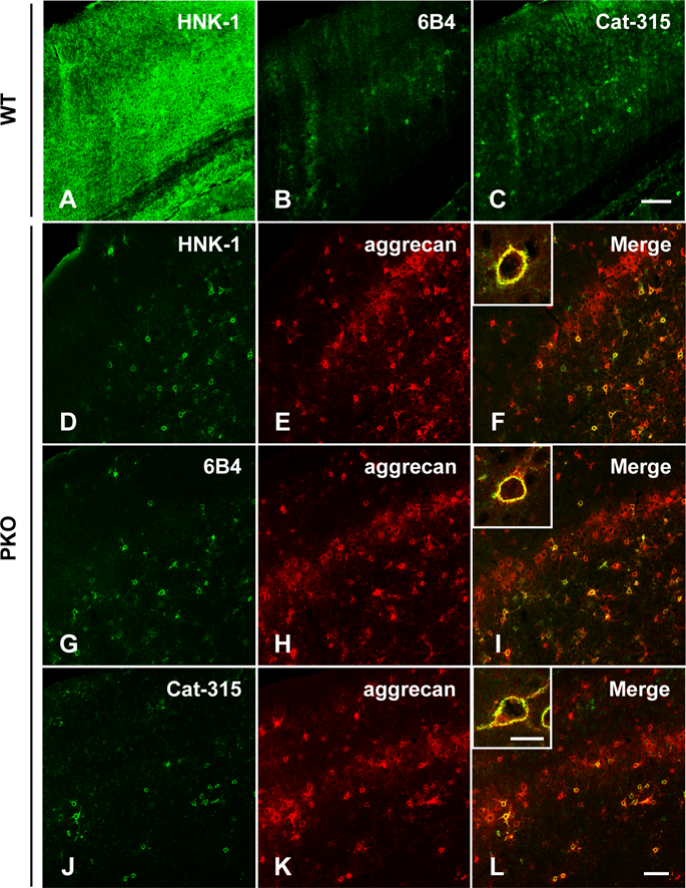
HNK-1 carbohydrate and aggrecan are expressed in the PNNs. Sagittal sections of cerebral cortex from 6-week-old WT (A-C) and PKO mice (D-L) were singly (for WT) or doubly (for PKO) immunostained with HNK-1 mAb (A, D), 6B4 mAb (B, G), or Cat-315 mAb (C, J), and aggrecan pAb (E, H, K). F, I and L are overlaid images. High magnification images of HNK-1-, 6B4- or Cat-315- and aggrecan-positive PNNs are shown in the insets. Scale bars, 200 μm (A-C), 100 μm (D-L) and 20 μm (insets).

Next, to examine whether the HNK-1 epitope on aggrecan was located in PNNs in mature PKO brains, 6-week-old mouse brain sections were immunostained using HNK-1, 6B4 or Cat-315 and anti-aggrecan antibodies. As we reported previously [31], PKO mice showed an overall reduced HNK-1 reactivity, but the HNK-1 signals in PNN-like pattern almost completely remained in the PKO brains (compare Fig 3A and 3D). The remaining HNK-1 epitope in the PKO brain was largely colocalized with aggrecan (Fig 3D–3F) and high magnification showed the colocalization in a typical PNN pattern (Fig 3F, inset). Staining of PKO sections with 6B4 and Cat-315 mAbs also showed the similar overlapped signals with aggrecan in PNN patterns (Fig 3G–3L). Furthermore, PNNs were clearly detected with 6B4 and Cat-315 mAbs compared with HNK-1 mAb, even in WT mouse brain sections (Fig 3A–3C), indicating that these two mAbs preferentially recognize the unique HNK-1 epitope in PNNs. Taken together, these results further confirmed that the HNK-1 epitope on aggrecan in PNNs is biosynthesized by an unknown pathway independent of GlcAT-P.

### HNK-1 epitope is synthesized by a novel glucuronyltransferase

Other than GlcAT-P, only one glucuronyltransferase, GlcAT-S, has been shown to biosynthesize the typical HNK-1 structure, HSO_3_-3GlcAβ1-3Galβ1-4GlcNAc-R [29]. To investigate whether the HNK-1 epitope in PNNs is biosynthesized by GlcAT-S, we generated GlcAT-S gene-deficient (SKO) mice as described in **Materials and Methods**. Briefly, construction of the targeting vector is schematically represented in Fig 1A. Eight ES clones among 356 tested showed the desired homologous recombination (Fig 1B). Two lines of SKO mice were generated from two independent ES clones. The heterozygotes were further backcrossed with C57BL/6 mice for more than 10 generations. The SKO mice were viable and fertile and their brains showed no obvious abnormalities (data not shown). As we reported previously, GlcAT-S is expressed in mouse kidney and the brain and is responsible for the non-sulfated HNK-1 carbohydrate in the kidney [44]. These non-sulfated HNK-1 carbohydrates detected with M6749 mAb, disappeared in the SKO kidney (Fig 1C).

**Fig 4:**
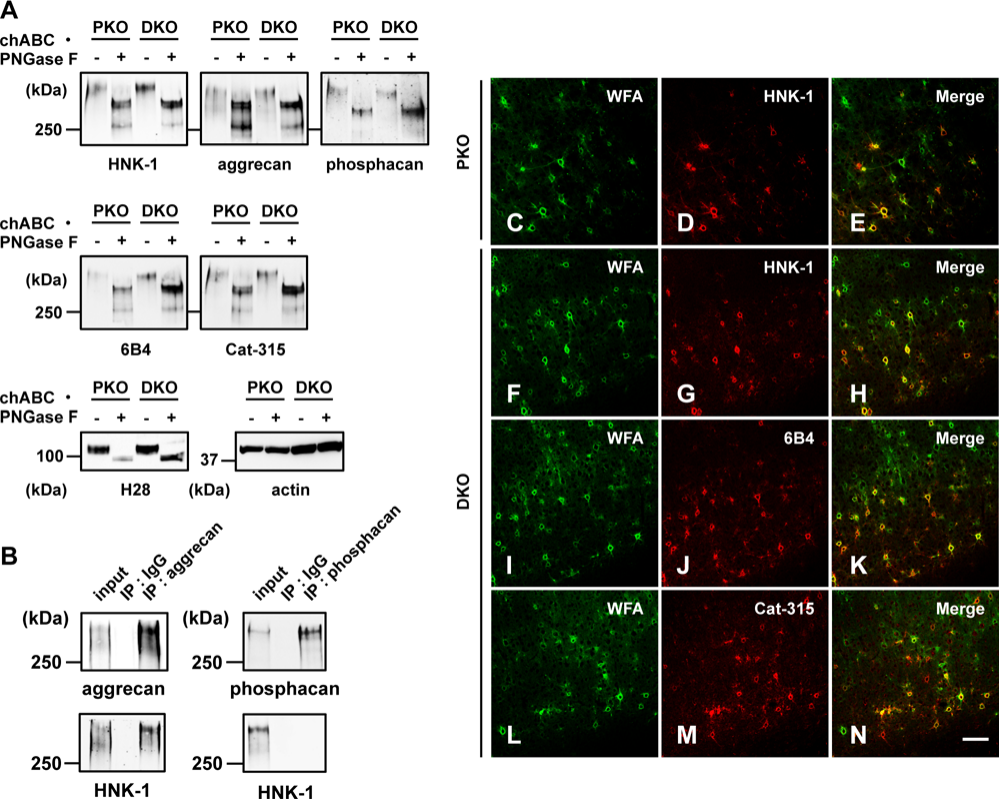
HNK-1 carbohydrate expressed in PKO and DKO mice. (A) Soluble fractions from 6-week-old GlcAT-P-deficient mice (PKO) and GlcAT-P and -S double deficient mice (DKO) were treated with chABC and PNGase F, subjected to SDS-PAGE and blotted using HNK-1 mAb, 6B4 mAb, Cat-315 mAb, aggrecan pAb, phosphacan pAb, H28 (NCAM mAb) or actin mAb. The effect of PNGase F treatment was confirmed by western blotting with H28 mAb. Actin was used as the loading control. (B) Using soluble fractions from 6-week-old DKO mouse brains (input), aggrecan and phosphacan were immunoprecipitated with aggrecan pAb (IP: aggrecan), normal rabbit IgG (IP: IgG) or phosphacan pAb (IP: phosphacan). The precipitated aggrecan and phosphacan were subjected to SDS-PAGE and western blotting with HNK-1, aggrecan and phosphacan antibodies. (C-N) Sagittal sections of cerebral cortex from 6-week-old PKO (C-E) and DKO mice (F-N) were double-immunostained with HNK-1 mAb, 6B4 mAb or Cat-315 mAb (*red*) and WFA lectin (*green*). Scale bar, 100 μm.

Next, the PKO and SKO mice were interbred to obtain double-knockout (DKO) mice. DKO mice were also viable and fertile. Brain soluble fractions from 6-week-old PKO and DKO mice were subjected to glycosidase digestion and western blot analyses. Reactivity with HNK-1 in DKO mice was not reduced compared with PKO mice (Fig 4A, *HNK-1 panel*). The molecular weight of the HNK-1 reactive band in DKO mice was also consistent with aggrecan rather than phosphacan (Fig 4A, *aggrecan and phosphacan panels*). In addition, the reactive band was detected using 6B4 and Cat-315 mAbs in the DKO mice (Fig 4A, *6B4 and Cat-315 panels*); the DKO and PKO mice showed similar results. The effect of PNGase F digestion and the amounts of loaded proteins were confirmed by immunoreactive bands with H28 (anti-NCAM mAb) and anti-actin mAb, respectively (Fig 4A, *H28 and actin panels*). These results indicate that aggrecan is the HNK-1 carrier protein in the 6-week-old DKO mouse brain. To further confirm this assumption, we immunoprecipitated aggrecan and phosphacan from brain soluble fractions of 6-week-old DKO mice and then immunoblotted the precipitated aggrecan and phosphacan using HNK-1, anti-aggrecan and anti-phosphacan antibodies. HNK-1 reactivity was observed on immunoprecipitated aggrecan but not on phosphacan (Fig 4B). Double-staining of brain sections with HNK-1, 6B4 or Cat-315 mAb and WFA lectin also showed the remaining HNK-1 reactivity in the PNNs of DKO mice (Fig 4F–4N), which was similar to PKO mice (Fig 3D, 3G and 3J and Fig 4C–4E). These results suggested a novel glucuronyltransferase, but neither GlcAT-P nor -S, was responsible for biosynthesis of the HNK-1 epitope in PNNs.

### HNK-1ST is involved in HNK-1 synthesis in COS-1 cells

**Fig 5:**
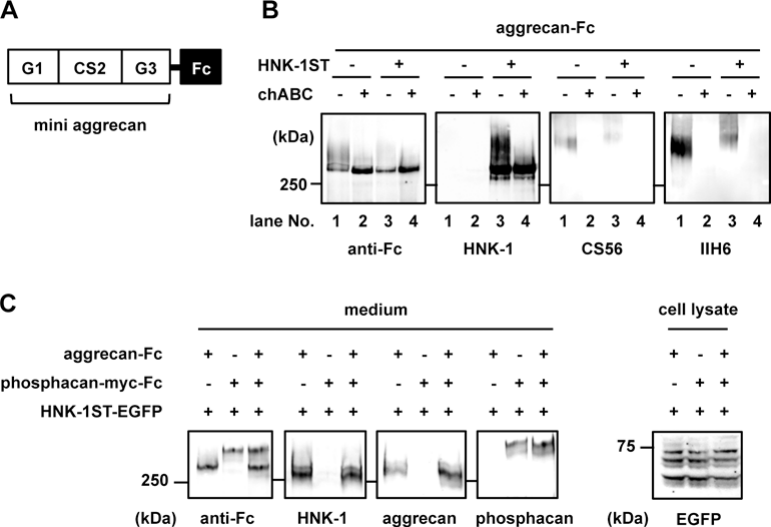
Expression of a novel HNK-1 epitope on aggrecan in COS-1 cells. (A) Schematic diagram of the Fc-tagged short aggrecan used in this study. (B) Aggrecan-Fc with or without HNK-1ST was heterologously expressed into COS-1 cells. Aggrecan-Fc in the culture medium was purified using a Protein G Sepharose column and digested with 100 μU/ml chABC and then blotted with anti-Fc pAb, HNK-1 mAb and anti-CS antibodies (CS56 and clone IIH6). (C) Aggrecan-Fc and/or phosphacan-myc-Fc were transiently expressed with HNK-1ST-EGFP into COS-1 cells. Aggrecan-Fc and phosphacan-myc-Fc in the culture medium were precipitated with ethanol and digested with 200 μU/ml chABC, subjected to SDS-PAGE and then blotted with anti-Fc pAb, HNK-1 mAb, aggrecan pAb and phosphacan pAb.

A possible glucuronyltransferase for biosynthesis of the HNK-1 epitope in PNNs is GlcAT-I that biosynthesizes glycosaminoglycans (GAGs) but displays extremely high homology to GlcAT-P (42.7% identity) [45]. GlcAT-I is the sole enzyme responsible for GlcA transfer for biosynthesis of the core tetrasaccharide structure of GAG, GlcAβ1-3Galβ1-3Galβ1-4Xyl, the so-called linkage region [46, 47]. Previous reports showed that overexpression of GlcAT-I elicits HNK-1 immunoreactivity [46] and HNK-1ST can act on the GAG linkage to suppress GAG elongation of thrombomodulin [48]. Based on these results, we assumed that GlcAT-I, working with HNK-1ST, was the most feasible candidate for biosynthesis of the HNK-1 epitope on aggrecan in the PNNs. To confirm this assumption, Fc-tagged short-form aggrecan (Fig 5A) was transiently expressed with or without HNK-1ST in COS-1 cells in which endogenous GlcAT-I but neither GlcAT-P nor GlcAT-S was expressed. Aggrecan-Fc was purified using Protein G Sepharose from culture medium and subjected to western blot analysis. When aggrecan-Fc was expressed alone, aggrecan-Fc was detected with anti-Fc antibody as a smear band over 300 kDa (Fig 5B *anti-Fc panel*, *lane 1*). When co-expressed with HNK-1ST, the level of the upper smear band of aggrecan-Fc was relatively reduced and instead a sharp lower band became prominent (Fig 5B, *anti-Fc panel*, *lane 3*). The upper smear band converged at the lower sharp band with chABC treatment (Fig 5B, *anti-Fc panel*, *lanes 2 and 4*), indicating the lower sharp band was a non-CS-bearing aggrecan. More importantly, the non-CS-bearing lower band was HNK-1-positive in an HNK-1ST dependent manner (Fig 5B, *HNK-1 panel*, *lane 3*). The reactivity with HNK-1 mAb was unaffected with PNGase F treatment (data not shown), which was consistent with the results in the PKO mouse brain (Fig 2). These results suggest the possibility that the unique HNK-1 epitope on aggrecan is synthesized by stepwise actions of GlcAT-I and HNK-1ST to create a unique sulfated type of GAG linkage region, HSO_3_-GlcAβ1-3Galβ1-3Galβ1-4Xyl.

Notably, the relative increase in the level of the non-CS-bearing lower band of aggrecan with HNK-1ST expression in Fig 5B suggests the addition of the HNK-1 epitope on aggrecan downregulates its CS chain biosynthesis. This was also supported by weaker immunoreactivities with anti-CS antibodies of aggrecan (CS56 and IIH6) in the presence of overexpressed HNK-1ST (Fig 5B, *CS56 and IIH6 panels*, *lanes 3)*. Quantification of the signal intensities in these blots revealed that CS56- and IIH6-immunoreactive signals of aggrecan-Fc in the presence of HNK-1ST were reduced to 40% and 44%, respectively, compared to those in the absence of HNK-1ST. In addition, we carried out a quantitative analysis of CS side chains using high-performance liquid chromatography. Recombinant aggrecan-Fc was expressed with or without HNK-1ST in COS-1 cells and purified from culture media. CS chains were prepared from the recombinant aggrecan-Fc by chABC treatment, and the amount and composition of CS-derived disaccharides were analyzed. While the disaccharide unit composition was unaffected, the total amount of CS on the aggrecan-Fc was decreased in the presence of HNK-1ST and the quantity ratio (ST(+)/ST(-) ratio) was similar to the value in the western blot analysis (Table 1). These results suggest that the synthesis of the HNK-1 epitope on the GAG linkage region inhibits its CS polymerization.

**Table 1:** Quantification of chondroitin sulfate on the purified recombinant aggrecan-Fc.

aggrecan-Fc	unsaturated disaccharide [pmol/ml]			
	ΔDi-0S	ΔDi-6S	ΔDi-4S	Total
**HNK-1ST(-)**	**120.9**	**145.5**	**318.4**	**584.8**
**HNK-1ST(+)**	**51.5**	**65.3**	**132.8**	**249.6**
**ST(+) / ST(-) ratio (%)**	**43%**	**45%**	**42%**	**43%**

The purified recombinant aggrecan-Fc expressed with or without HNK-1ST was digested with chABC. The digests were derivatized with a fluorophore, 2-aminobenzamide, and then analyzed by anion-exchange high-performance liquid chromatography. Each value was normalized by the amount of aggrecan-Fc. ΔDi-0S, Δ^4,5^HexUAα1-3GalNAc; ΔDi-6S, Δ^4,5^HexUAα1-3GalNAc (6-*O*-sulfate); ΔDi-4S, Δ^4,5^HexUAα1-3GalNAc (4-*O*-sulfate). The lowest row shows the quantity ratio of chondroitin sulfate on aggrecan-Fc with or without HNK-1ST expression.

As shown in Fig 4B, the HNK-1 epitope in PNNs was specifically expressed on aggrecan but not on phosphacan. To examine whether this specific HNK-1 expression is also observed in COS-1 cells, phosphacan-myc-Fc and/or aggrecan-Fc were transiently expressed with HNK-1ST. As shown in Fig 5C, HNK-1 immunoreactivity was not detected on phosphacan-myc-Fc but on the aggrecan-Fc. This result implies that HNK-1ST preferentially recognizes aggrecan as an acceptor rather than phosphacan, but the mechanism underlying this protein-selective modification is unclear at present.

### Identification of the glycan structure of the HNK-1 epitope on aggrecan

**Fig 6:**
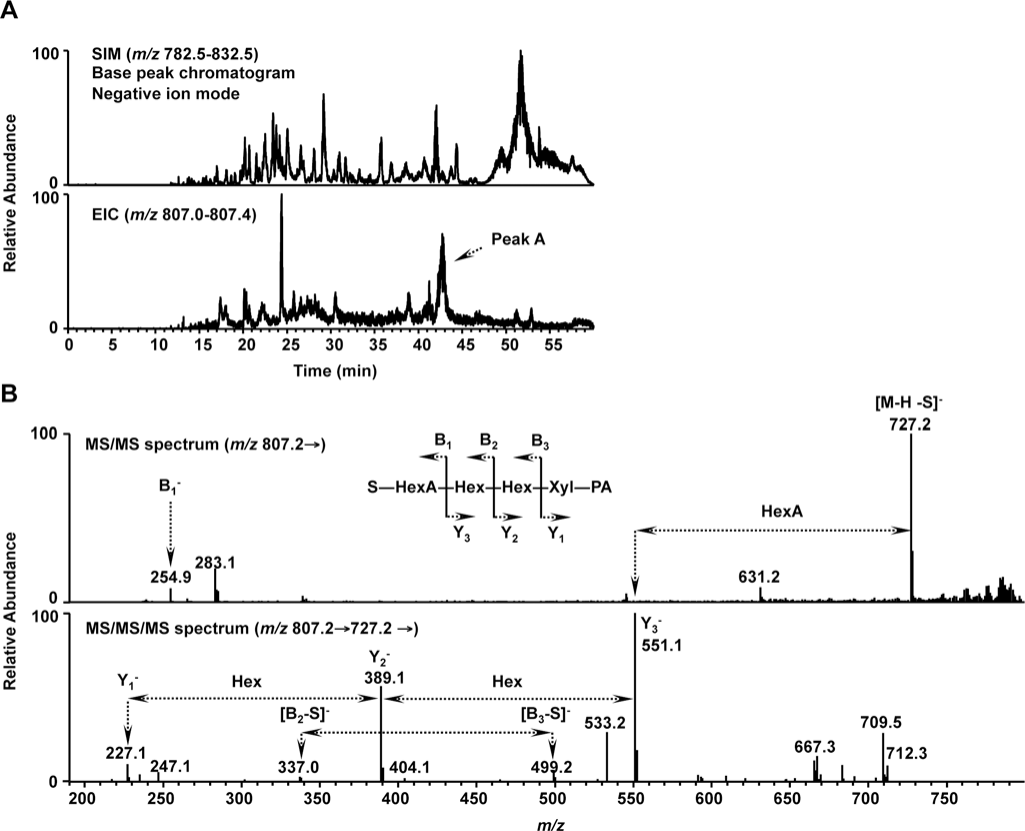
LC/MS^n^ structural analysis of HNK-1 epitope on aggrecan. (A) PA-labeled *O*-linked glycans were prepared from aggrecan-Fc co-expressed with HNK-1ST in COS-1 cells. The base peak chromatogram of the glycans was obtained using selected ion monitoring (SIM) (*m/z* 782.5–832.5) in the negative ion mode (*upper panel*). An extracted ion chromatogram (EIC) of the ion at *m/z* 807.0–807.4 (*lower panel*). (B) MS/MS spectra (*upper panel*) of the ion [M-H]^-^ (*m/z* 807.2) detected in peak A and MS/MS/MS spectra (*lower panel*) of the predominant product ion (*m/z* 727.2) in the MS/MS. S, sulfate group; HexA, hexuronic acid; Hex, hexose; Xyl-PA, xylose labeled with 2-aminopyridine.

To further confirm that the sulfated linkage region is synthesized on aggrecan, aggrecan-Fc was co-expressed with HNK-1ST and purified from COS-1 cells. HNK-1-positive aggrecan was treated with chABC and PNGase F and then released *O*-glycans were PA-labeled and analyzed using LC/MS^n^. Fig 6A upper panel shows the base peak chromatogram obtained using SIM (*m/z* 782.5–832.5) in the negative ion mode. The major precursor ions in the mass spectra were automatically subjected to data-dependent collision-induced dissociation-MS/MS and MS/MS/MS. The extracted ion chromatogram (EIC) from *m/z* 807.0 to 807.4 is shown in Fig 6A lower panel. The singly charged ion (*m/z* 807.2) corresponding to PA-labeled HSO_3_-GlcA-Gal-Gal-Xyl was detected in Peak A of the EIC. In the MS/MS spectrum of this ion, the product ion at *m/z* 727.2 corresponded to [M-H-S]^-^ and the ion at *m/z* 254.9 corresponded to B_1_^-^ (Fig 6B, *upper panel*). In the MS/MS/MS spectrum acquired from product ion (*m/z* 727.2) in the MS/MS spectrum, the ions at *m/z* 551.1, 389.1 and 227.1 corresponded to Y_3_^-^, Y_2_^-^ and Y_1_^-^, respectively. The ions at *m/z* 499.2 and 337.0 corresponded to [B_3_-S]^-^ and [B_2_-S]^-^, respectively. These results indicated the sulfate group was attached to the HexA-Hex-Hex-Xyl and most likely to HexA. From these results, we concluded the sulfated tetrasaccharide, HSO_3_-GlcA-Gal-Gal-Xyl, was expressed on aggrecan-Fc, the probable HNK-1 epitope expressed on aggrecan in PNNs.

## Discussion

**Fig 7:**
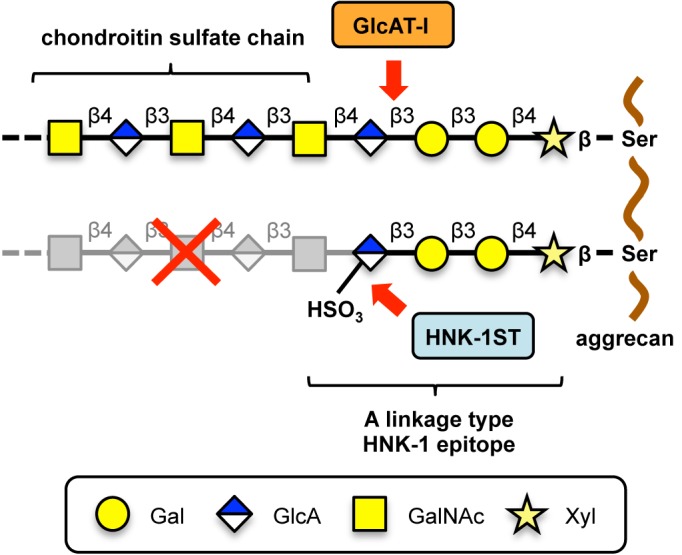
Biosynthetic model for HNK-1 epitope on aggrecan in PNNs. GlcAT-I is responsible for synthesis of a linkage region of GAG, which is usually further elongated into a long GAG chain (e.g., CS chain). HNK-1ST transfers a sulfate group to GlcA of the linkage region of aggrecan, which likely results in the expression of the linkage type HNK-1 epitope, HSO_3_-GlcA-Gal-Gal-Xyl, in PNNs. The expression of the HNK-1 epitope on aggrecan suppresses the CS polymerization that starts from GlcA of the linkage region.

In this study, by analyzing double knockout mice for GlcAT-P (*B3gat1*) and GlcAT-S (*B3gat2*) both of which are involved in the canonical HNK-1 biosynthesis, we demonstrated that a unique HNK-1 structure is expressed on aggrecan in PNNs. Using COS-1 cells in which neither GlcAT-P nor GlcAT-S is expressed, we identified the unique HNK-1 epitope as a sulfated type of GAG linkage region synthesized on aggrecan in an HNK-1ST dependent manner. Therefore, we named this structure a linkage type HNK-1 epitope to be distinguished from classical HNK-1. Furthermore, expression of such unique HNK-1 epitope on aggrecan suppresses its CS biosynthesis (Fig 7).

To date, the HNK-1 epitope has been shown to be expressed on *N*-linked glycans or *O*-mannosylated glycans in the CNS [32, 42, 43, 49] and 6B4 and Cat-315 mAbs, which are widely used to stain PNNs, were believed to recognize the *N*-linked or *O*-mannosylated types of HNK-1 epitopes [37, 39, 40, 41]. In the present study, we demonstrated that a third type of HNK-1 epitope is expressed on the GAG linkage region of aggrecan after formation of PNNs and 6B4 and Cat-315 mAbs have higher specificity for that type of HNK-1 epitope (Fig 2). Considering the reactivity of these mAbs to *O*-mannosylated type of HNK-1 in the developing brain [37, 39, 40, 41] (Fig 2A), epitopes of 6B4 and Cat-315 mAbs are likely switched during development from *O*-mannosylated HNK-1 on phosphacan to the linkage region type of HNK-1 on aggrecan. Several antibodies recognize the HNK-1 epitope [24–26]; however, we suggest that 6B4 and Cat-315 are better probes than HNK-1 mAb to detect the linkage type HNK-1 epitope in PNNs (Fig 3A, 3B and 3C). Recently, the serum of peripheral neuropathy patients having 6B4 or Cat-315 type autoantibodies showed higher treatment resistance than patients with general type of HNK-1 autoantibodies [50]. Difference in specificity of HNK-1-related mAbs could be a good indicator for severity of demyelinating diseases.

PNNs are expressed around GABAergic interneurons, especially parvalbumin expressing cells [10, 11, 13]. GABAergic interneurons are highly heterogeneous [51]. For example, more than 20 classes of interneurons have been identified in the hippocampus and neocortex, each with distinctive spatial and temporal capabilities to influence cortical circuits [52, 53]. HNK-1 positive PNNs largely colocalized with aggrecan or WFA but PNNs expressing aggrecan or WFA were not always HNK-1-positive (Figs 3D–3L and 4C–4N), indicating that expression of the linkage type HNK-1 epitope is distinctly regulated around a subset of neurons. In addition, the number of Cat-315-positive PNNs changes in an activity-dependent manner in the rat superior olivary complex and the mouse barrel cortex [21, 54], and 6B4 and Cat-315-positive PNNs were prominently reduced in the age-dependent neurodegeneration model mouse brain [55]. These findings suggest that the linkage type HNK-1 epitope in PNNs is functionally involved in neural plasticity. Therefore, to better understand the role of HNK-1 in PNNs, identifying the cell types among the GABAergic interneurons that express this epitope is important.

Our structural analysis suggests that GlcAT-I, the sole enzyme identified for synthesis of the GAG linkage region to date, might be involved in the linkage type HNK-1 epitope on aggrecan in PNNs. Previous findings showing overexpression of GlcAT-I evoked HNK-1 immunoreactivity [46] and the sulfated linkage region was detected in human urine [56, 57] further supports our hypothesis that GlcAT-I is the enzyme responsible for the synthesis of the unusual HNK-1 on aggrecan. However, directly demonstrating its involvement is difficult because GlcAT-I (*B3gat3*)-knockout mice show embryonic lethality at a very early stage [58]. In addition, it is also difficult to demonstrate the contribution of GlcAT-I by its knockdown targeting to PNN-forming interneurons because the HNK-1 epitope is expressed in a subpopulation of PNN-forming interneurons, which would make it hard to distinguish the signal reduction in HNK-1 by GlcAT-I silencing from the original feature as a non-HNK-1-expressing PNN.

We also demonstrated that heterologous expression of HNK-1ST is required for synthesis of the linkage type HNK-1 epitope. Recently, it was shown that HNK-1ST can transfer a sulfate group to steroid hormones to regulate estrogen signaling [59]. Furthermore, we reported that HNK-1ST negatively regulates biosynthesis of *O*-mannosylated laminin-binding glycan essential for muscular functions [60]. These results indicate that HNK-1ST is a versatile enzyme and transfers a sulfate group to more substrates than expected.

We showed that HNK-1ST suppressed CS biosynthesis by synthesizing a HNK-1 epitope on the linkage region of aggrecan (Fig 5B and Table 1). Considering the importance of the CS chain or its degree of sulfation for neural plasticity such as ocular dominance plasticity [16, 23, 61, 62], the linkage type HNK-1 epitope may indirectly regulate neural plasticity by reducing the number of CS chains. In PNN-formed 6-week-old PKO or DKO mouse brains, even after the linkage type HNK-1 epitope was expressed, aggrecan still showed the smear band pattern, indicating that aggrecan possesses CS chains and simultaneously the linkage type HNK-1 epitope. These results suggest that a limited number of CS chain attachment sites (i.e., several specific sites) on aggrecan are sulfated and negatively regulated by HNK-1ST. A recent report showed that synthesis of the CS chain is regulated by phosphorylation of the Xyl residue in the linkage region [63], thus phosphorylation of Xyl may help HNK-1ST recognize a linkage region.

In conclusion, in the present study we discovered a new aspect of the HNK-1 epitope. These results shed light on the distinct *in vivo* roles of three homologous glucuronyltransferases (GlcAT-P, -S, -I) for HNK-1 synthesis. Further studies should aim to clarify how the linkage type HNK-1 epitope is involved in neural plasticity and GAG synthesis in PNNs in the brain.
